# Streamlining management in thoracic trauma: radiomics- and AI-based assessment of patient risks

**DOI:** 10.3389/fsurg.2024.1462692

**Published:** 2024-10-24

**Authors:** Ashraf F. Hefny, Taleb M. Almansoori, Darya Smetanina, Daria Morozova, Roman Voitetskii, Karuna M. Das, Aidar Kashapov, Nirmin A. Mansour, Mai A. Fathi, Mohammed Khogali, Milos Ljubisavljevic, Yauhen Statsenko

**Affiliations:** ^1^ Department of Surgery, College of Medicine and Health Sciences, United Arab Emirates University, Al Ain, United Arab Emirates; ^2^ Department of Radiology, College of Medicine and Health Sciences, United Arab Emirates University, Al Ain, United Arab Emirates; ^3^ Medical Imaging Platform, ASPIRE Precision Medicine Research Institute Abu Dhabi, Al Ain, United Arab Emirates; ^4^ Department of Family Medicine, Ambulatory Health Services, SEHA, Al Ain, United Arab Emirates; ^5^ Department of Surgery, Ain Shams University, Cairo, Egypt; ^6^ Institute of Public Health, College of Medicine and Health Sciences, United Arab Emirates University, Al Ain, United Arab Emirates; ^7^ Department of Physiology, College of Medicine and Health Sciences, United Arab Emirates University, Al Ain, United Arab Emirates; ^8^ Neuroscience Platform, ASPIRE Precision Medicine Research Institute Abu Dhabi, Al Ain, United Arab Emirates

**Keywords:** blunt chest trauma, injury severity score (ISS), abbreviated injury scale (AIS), radiomics, chest CT scans, regression models, risk factors, patient management

## Abstract

**Background:**

In blunt chest trauma, patient management is challenging because clinical guidelines miss tools for risk assessment. No clinical scale reliably measures the severity of cases and the chance of complications.

**Aim:**

The objective of the study was to optimize the management of patients with blunt chest trauma by creating models prognosticating the transfer to the intensive care unit and in-hospital length of stay (LOS).

**Methods:**

The study cohort consisted of 212 cases. We retrieved information on the cases from the hospital’s trauma registry. After segmenting the lungs with Lung CT Analyzer, we performed volumetric feature extraction with data-characterization algorithms in PyRadiomics.

**Results:**

To predict whether the patient will require intensive care, we used the three groups of findings: ambulance, admission, and radiomics data. When trained on the ambulance data, the models exhibited a borderline performance. The metrics improved after we retrained the models on a combination of ambulance, laboratory, radiologic, and physical examination data (81.5% vs. 94.4% Sn). Radiomics data were the top-accurate predictors (96.3% Sn). Age, vital signs, anthropometrics, and first aid time were the best-performing features collected by the ambulance service. Laboratory findings, AIS scores for the lower extremity, abdomen, head, and thorax constituted the top-rank predictors received on admission to the hospital. The original first-order kurtosis had the highest predictive value among radiomics data. Top-informative radiomics features were derived from the right hemithorax because the right lung is larger. We constructed regression models that can adequately reflect the in-hospital LOS. When trained on different groups of data, the machine-learning regression models showed similar performance (MAE/ROV≈8%). Anatomic scores for the body parts other than thorax and laboratory markers of hemorrhage had the highest predictive value. Hence, the number of injured body parts correlated with the case severity.

**Conclusion:**

The study findings can be used to optimize the management of patients with a chest blunt injury as a specific case of monotrauma. The models we built may help physicians to stratify patients by risk of worsening and overcome the limitations of existing tools for risk assessment. High-quality AI models trained on radiomics data demonstrate superior performance.

## 1 Introduction

Each year, traumatic injuries cause more than 5 million deaths globally with blunt chest injury - as a monotrauma - accounting for nearly 25% of deaths (1, 2). The most common reasons for blunt chest trauma (BCT) are motor vehicle accidents (MVA), falls, violence, and blast injuries. The mortality rate is high: according to literature findings, it ranges from 4 to 20% (3–5).

In BCT, patient management is challenging because clinical guidelines miss tools for risk assessment. Typically, risk assessment is based on the following clinical signs: orthopnea, haemodynamic instability, respiratory problems, and high injury scores (6, 7). Older age and multiple rib fractures suggest admission to the Intensive Care Unit (ICU). Still, some prognostic factors may not be apparent, which justifies the practical necessity to optimize patient management by introducing validated risk scales. Severe symptoms may develop within 72 h, therefore close monitoring of BCT patients is required (6). Early ICU admission may also reduce the in-hospital length of stay (LOS), thus improving patients’ well-being and maximizing the use of hospital resources (8, 9).

For risk stratification in BCT, healthcare professionals use two major groups of clinical scales (10). The scales of the first group measure the level of consciousness. However, a study showed that the most common instrument for guiding immediate medical care, monitoring hospitalized patients, and tracking their level of consciousness - Glasgow coma scale (GCS) - inaccurately reflects mortality and ICU admission (11). The scales of the other group are anatomic scoring systems classifying each injury by body region, they evaluate the physical condition of the whole body and/or its parts. The final severity score is the sum of the scale values for all anatomic regions, for instance, injury severity score (ISS), new injury severity score (NISS), and abbreviated injury scale (AIS). The weaknesses of these scoring systems are multifold. First, the estimation of the total effect of a trauma with a simple adding model is non-reliable. Second, these scores do not consider age and pre-existing chronic pathology. Third, they prognosticate short-term outcomes of a trauma (10), but the long-term prognosis is not accurate (12). In patients with isolated rib fractures, the AIS scores prognosticate respiratory complications with high sensitivity and low specificity: 94.43% vs. 18.79% (13).

Radiological scores have been mainly used to analyze chest computed tomography (CT) findings in tuberculosis, COVID-19-associated, and other pneumonia, but they are also required in BCT for efficient risk stratification (14–17). The existing chest trauma scores combine clinico-demographic parameters with CT findings. For example, the thoracic trauma severity score is calculated from the data on rib fractures, lung contusions, PaO2/FiO2 ratio, age, and pleural involvement (18). For risk assessment in pulmonary diseases, bioengineers constructed structure-function association models reflecting the level of lung impairment (19–22). Despite the availability of clinical and radiologic scores for assessing the severity of cases, no clinical tool can reliably measure the risks of complications in BCT.

## 2 Objectives

The primary objective is to optimize the management of the BCT monotrauma patients. *The working hypothesis of the current study is that*, a combined analysis of the ambulance and admission data allows us to build high-accurate classifications and regressions to prognosticate trauma outcomes. The predictors may include trauma anamnesis, clinico-demographic parameters, laboratory findings, and radiomics. With feature engineering, we will identify the top risks of patient worsening and build reliable models to aid physicians in decision-making.

To reach the aim, we formulated the following *specific objectives*:

1. Study hospital statistics on the transfer of BCT patients to ICU department.
2. Use ambulance and admission data to create a prediction model for ICU hospitalization.
3. Train a regression model to prognosticate in-hospital length of stay (LOS) from demographics, clinical risk scores, laboratory data, and radiological findings.

## 3 Materials and methods

### 3.1 Study cohort

We analyzed the cases of admission to a community-based hospital with BCT (Al Ain Hospital, United Arab Emirates) for 3 consecutive years. The inclusion criteria were inpatient admission and confirmed diagnosis of BCT (ICD-9 959.11; ICD-10 S29.8XXA). Penetrating chest trauma was an exclusion criterion. In total, 212 cases were selected for the analysis. The study sample included 184 males and 28 females aged 31±15 and 33±18 years, respectively.

### 3.2 Study data

Retrospectively, we collected the patient data. The injury details were retrieved from the trauma registry of the hospital. The missing information was supplemented by manual retrieval from patients’ electronic medical records. The data were classified into three groups. The first group comprised the ambulance data: trauma anamnesis, demographics, anthropometrics, vital signs, and GCS. The second group included admission findings: the results of laboratory (biochemical) tests, physical examination, reported CT findings, and anatomical scores. Finally, the third group was the radiomics data retrieved from the early chest CT with computer frameworks. See Table 1 for the full list of features taken into analysis.

**Table 1:** Groups of data taken for analysis.

1. Ambulance data
**Numerical features:** Age, BMI, Body, Heart rate, Height, Respiratory rate, Soft tissue injury, Systolic blood pressure, Temperature, First aid time, O_2_ saturation, Glasgow Coma Scale
**Categorical features:** Mechanism of trauma, Occupant seat in MVC, Place of injury, Sex, Transfer from another facility, Type of MVC, Work related
2. Physical examination, lab & radiologic findings on admission
**Numerical features:** AIS Abdomen, AIS External, AIS Face, AIS Head, AIS Lower Extremity, AIS Spine, AIS Thorax, AIS Upper Extremity, Hemoglobin, Haematocrit,
**Categorical features:** Abdomen injury, Diaphragm injury, First and/or second rib fracture, Flail chest, Great vessel injury, Hemothorax, Head injury, Multiple ribs fracture, Single rib fracture, Spine injury, Sternum fracture, Surgical emphysema, Upper extremity injury,
3. Radiomics data retrieved from early chest CT
**firstorder:** 10Percentile, 90Percentile, Energy, Entropy, InterquartileRange, Kurtosis, Maximum, MeanAbsoluteDeviation, Mean, Median, Minimum, Range, RobustMeanAbsoluteDeviation, RootMeanSquared, Skewness, TotalEnergy, Uniformity, Variance
**glcm:** Autocorrelation, ClusterProminence, ClusterShade, ClusterTendency, Contrast, Correlation, DifferenceAverage, DifferenceEntropy, DifferenceVariance, Id, Idm, Idmn, Idn, Imc1, Imc2, InverseVariance, JointAverage, JointEnergy, JointEntropy, MCC, MaximumProbability, SumAverage, SumEntropy, SumSquares
**gldm:** DependenceEntropy, DependenceNonUniformity, DependenceNonUniformityNormalized, DependenceVariance, GrayLevelNonUniformity, GrayLevelVariance, HighGrayLevelEmphasis, LargeDependenceEmphasis, LargeDependenceHighGrayLevelEmphasis, LargeDependenceLowGrayLevelEmphasis, LowGrayLevelEmphasis, SmallDependenceEmphasis, SmallDependenceHighGrayLevelEmphasis, SmallDependenceLowGrayLevelEmphasis
**glrlm:** GrayLevelNonUniformity, GrayLevelNonUniformityNormalized,GrayLevelVariance, HighGrayLevelRunEmphasis, LongRunEmphasis, LongRunHighGrayLevelEmphasis, LongRunLowGrayLevelEmphasis, LowGrayLevelRunEmphasis, RunEntropy, RunLengthNonUniformity, RunLengthNonUniformityNormalized, RunPercentage, RunVariance, ShortRunEmphasis, ShortRunHighGrayLevelEmphasis, ShortRunLowGrayLevelEmphasis
**glszm:** GrayLevelNonUniformity, GrayLevelNonUniformityNormalized, GrayLevelVariance, HighGrayLevelZoneEmphasis, LargeAreaEmphasis, LargeAreaHighGrayLevelEmphasis, LargeAreaLowGrayLevelEmphasis, LowGrayLevelZoneEmphasis, SizeZoneNonUniformity, SizeZoneNonUniformityNormalized, SmallAreaEmphasis, SmallAreaHighGrayLevelEmphasis, SmallAreaLowGrayLevelEmphasis, ZoneEntropy, ZonePercentage, ZoneVariance
**ngtdm:** Busyness, Coarseness, Complexity, Contrast, Strength
**shape:** Elongation, Flatness, LeastAxisLength, MajorAxisLength, Maximum2DDiameterColumn, Maximum2DDiameterRow, Maximum2DDiameterSlice, Maximum3DDiameter, MeshVolume, MinorAxisLength, Sphericity, SurfaceArea, SurfaceVolumeRatio, VoxelVolume

For correlating radiological data with clinical outcomes, we resorted to radiomics which uses advanced mathematical analysis to enhance the data available to clinicians (23). We applied a two-step approach to extract radiomics features from medical images. To start with, we used a Lung CT Analyzer extension of 3D Slicer application to segment the lungs. The extension utilizes a fully convolutional network (U-Net) which yields precise segmentation (24). U-net (R231) is a deep-learning model from this software, and it automatically delineates the right and left hemithorax - the cavities lateral to the mediastinum (25). After applying segmentation masks to CT scans, we performed volumetric feature extraction with data-characterization algorithms implemented in the PyRadiomics package (26).

The radiomics package contains several categories of functions. *First-order statistics* describe the distribution of voxel intensities within the image region defined by the mask through commonly used and basic metrics. *Gray Level Co-occurrence Matrix* (GLCM) describes the second-order joint probability function of the image region. *Gray Level Dependence Matrix* (GLDM) quantifies gray level dependencies in the image. *Gray Level Run Length Matrix* (GLRLM) quantifies gray level runs defined as the length in some consecutive pixels of the same gray level. *Gray Level Size Zone* (GLSZM) quantifies gray level zones in an image. *Neighbouring Gray Tone Difference Matrix* (NGTDM) quantifies the difference between the gray value of a pixel/voxel and the average gray values of surrounding pixels/voxels. The last group of functions is called ’*shape*’ since it describes the two-dimensional size and shape of the region of interest. The radiomics features are independent of the distribution of gray level intensity, therefore they are calculated only for non-derived images and masks.

### 3.3 Study methodology

*To address the first task*, we explored the differences in trauma circumstances, clinico-demographic variables, laboratory and radiologic findings between ICU and non-ICU patients. A regression-based imputation was used to handle the missing data. If the percentage of the missing data exceeded 15%, we deleted the variable from the analysis. For other cases, we used a multiple linear regression model to predict the values. The Shapiro–Wilk test was used to assess data distribution. To compare normally-distributed variables, we used parametric tests: Student’s t-test, and the Pearson correlation coefficient. The variables that do not show Gaussian distribution, were analyzed with non-parametric Mann–Whitney and Wilcoxon signed-rank tests. Chi-square was used to examine the differences between categorical variables. We explored the following features of ICU and non-ICU cohorts: trauma circumstances, clinico-demographic, radiologic, and laboratory findings on admission.

*The second task was multifold*. First, we extracted radiomics from chest CT images. For this, we acquired masks of hemithoraces and calculated the radiomics data for each hemithorax. Second, we investigated clinical, demographic, radiomics and laboratory correlates of ICU admission. Third, we trained classification algorithms to predict transfer to ICU with tree-based machine learning (ML) methods such as XGBoost, CatBoost, Random Forest and Decision Tree. Feature importance was calculated with specific API functions of the LGBM framework. The optimal classification model was selected by calculating sensitivity (Sn), specificity (Sp), F1-score (F1), and area under the curve (AUC) in receiver-operating characteristic diagrams (ROC). Finally, we compared the accuracy of the models trained on different groups of predictors and their combinations.

*Working on the third task*, we employed the following regressors: DecisionTree, RandomForest, XGB, LGBM, and CatBoost. Regressor parameters were set to default values. To raise model accuracy, we explored feature importance of the predictors described in Subsection 3.2. The feature engineering technique was the same as in the previous task. Then, we calculated the final performance of the regression models reflecting the in-hospital LOS from the three groups of predictors: ambulance data, findings reported on admission and radiomics data retrieved from early chest CT. The ratio of mean absolute error (MAE) to the range of values (ROV) was the metrics of success.

### 3.4 Acquiring and preprocessing of CT findings

Radiotechicians used different protocols to acquire lung CT scans, most of which had 512×512 image resolution. The slice thickness ranged from 1 to 1.25 mm. To ensure uniformity of data across different machines with varying acquisition protocols, we used a standard approach to data preprocessing (27, 28).

Before radiomics feature extraction, a bioengineer did the computations in several steps. The first step was *image resampling*. To standardize the spatial resolution, we resembled all images to a uniform voxel size of 1×1×1 mm. It was performed with linear interpolation, ensuring consistency of spatial dimensions across all images.

As the second step of data preprocessing, we performed *normalization of signal intensity*. The intensity varied due to the difference in the settings of acquisition protocols. To reduce the variations, the intensity values of all images were normalized to a consistent range and clipped to the interval from −1,000 to 400 Hounsfield units. This allowed us to exclude extreme outliers and focus on the features relevant to the study. Then, the data were linearly rescaled to the range of values from 0 to 1.

*Histogram matching* was the final step of data preprocessing. It helped us to harmonize the distributions of intensities across the images by aligning intensity with a reference. Thus, we ensured consistency in data patterns and minimized a potential bias due to varying imaging protocols.

## 4 Results

### 4.1 Characteristics of study cohort

Transfer to the ICU occurred in only 26% of the cases admitted to the hospital. On average, the patients who went to ICU were significantly younger than those who did not stay in the intensive care department (27.86±14.72 vs. 32.76±16.68, p=0.026). All the cases did not meet the criteria for polytrauma since only the AIS thorax was greater than 2, and the severity index was lower in the other parts of the body (29).

The majority of traumas occurred in the street (66%) and they were more severe. The home traumas were significantly milder. A prominent difference was observed among ICU and non-ICU patients who received BCT at home (1 vs. 19 cases respectively, p=0.023). The majority of injuries occurred as a result of traffic accidents and falls from the height of over one meter (69% and 18%, respectively). In case of MVC, ICU and non-ICU patients received a significant portion of traumas in car collisions (See Table 2). Drivers of motor vehicles represented a majority of patients with injury to the chest (30%).

**Table 2:** Statistics on trauma circumstances.

Parameter	Total	ICU		Non-ICU		$p_{2-3}$
	$n_{1}=212$	$n_{2}=56$		$n_{3}=156$		
Place of injury						
Work place	39	10	18%	29	19%	0.905
Desert	8	1	2%	7	4%	0.365
Home	20	1	2%	19	12%	**0.023**
Street	139	42	75%	97	62%	0.084
Others	6	2	4%	4	3%	0.701
Mechanism of trauma						
Motor vehicle collisions	146	44	79%	102	65%	0.068
Fall from less than 1 meter	9	2	4%	7	4%	0.774
Fall from more than 1 meter	38	9	16%	29	19%	0.675
Hit by falling object	1	0	0%	1	1%	0.556
Others	18	1	2%	17	11%	**0.037**
Type of motor vehicle collisions						
Car collisions	108	32	57%	76	49%	0.281
Motorcycle	7	3	5%	4	3%	0.319
Bicycle	6	1	2%	5	3%	0.587
Pedestrian	24	8	14%	16	10%	0.416
Occupied seat in MVC						
Driver seat	64	16	29%	48	31%	0.760
Passenger front seat	30	10	21%	20	13%	0.356
Passenger back seat	13	6	11%	7	4%	0.097

Significant (*p* < 0.05) differences between ICU and non-ICU cohorts are marked in bold font.

Categorical data are expressed as n (%); continuous variables as mean ± SD.

ICU patients had a notably higher heart rate compared to non-ICU group (100.11±27.54 vs. 89.58±19.87, p=0.008). Both groups had a heart rate within the normal range for adults. A respiratory rate was significantly faster in patients requiring critical care (23.2±6.42). Both groups had an elevated breathing rhythm: above 16 breaths per minute.

Laboratory findings were within the normal range for both groups of patients (See Table 3). The haemoglobin level was considerably lower in the ICU cohort than in non-ICU patients (131.8±23.95 vs. 143.38±19.63). The percentage of hematocrit was notably higher in the non-ICU cohort. The statistical difference could occur due to a greater blood loss in critical patients.

**Table 3:** Clinicodemographic characteristics of patients with BCT.

Parameter	Total		ICU		Non-ICU		$p_{2-3}$
	$n_{1}=212$		$n_{2}=56$		$n_{3}=156$		
Demographics and anthropometrics							
Males	184	87%	46	82%	138	88%	0.149
Age, years	31.41±16.3		27.86±14.72		32.76±16.68		**0.026**
BMI, kg/m^2^	25.38±6.87		25.35±5.46		25.4±7.33		0.765
Vital signs							
Systolic blood pressure, mm Hg	131±23		127.21±25.64		132.16±21.43		0.082
Heart rate, per min	92.00±23.00		100.11±27.54		89.58±19.87		**0.008**
Respiratory rate, per min	21.00±5.00		23.20±6.42		20.57±4.49		**0.001**
O2 saturation, %	97.81±3.07		97.02±4.75		98.09±2.13		0.812
Body temperature, C	36.71±0.29		36.68±0.37		36.72±0.25		0.812
Laboratory findings							
Hemoglobin, g/L	140.33±21.42		131.80±23.95		143.38±19.63		**0.002**
Hematocrit, %	41.00±6.00		0.39±0.07		0.42±0.05		**0.002**
Clinical scores							
GCS	14.09±2.59		12.54±3.79		14.65±1.67		**<0.001**
ISS	16.52±8.59		22.11±9.86		14.52±7.12		**<0.001**
NISS	19.04±9.78		25.25±10.73		16.81±8.40		**<0.001**
AIS Head	0.62±1.21		1.16±1.72		0.43±0.89		**0.009**
AIS Face	0.26±0.59		0.45±0.76		0.19±0.50		**0.017**
AIS Neck	0.05±0.22		0.02±0.13		0.06±0.24		0.182
AIS Thorax	3.00±0.91		3.12±0.79		2.96±0.95		0.367
AIS Abdomen	0.48±1.02		0.91±1.42		0.33±0.78		**0.007**
AIS Spine	0.63±0.94		0.88±1.03		0.54±0.89		**0.028**
AIS Upper Extremity	0.65±1.00		0.77±1.06		0.61±0.97		0.358
AIS Lower Extremity	0.73±1.11		1.09±1.19		0.60±1.05		**0.004**
AIS External	0.03±0.19		0.07±0.32		0.01±0.11		0.084
CT findings reported by radiologist							
Hemothorax	27	13%	11	20%	17	11%	0.098
Pneumothorax	76	36%	24	43%	52	33%	0.203
Single rib fracture	16	8%	3	5%	16	10%	0.329
Multiple ribs fracture	69	33%	23	41%	46	29%	0.114
Soft tissue injury	65	31%	15	27%	52	33%	0.414
Sternum fracture	6	%	2	4%	8	5%	0.641
Surgical emphysema	18	9%	9	16%	9	6%	**0.031**
Injuries of other body parts							
Head	114	54%	34	62%	80	51%	0.128
Neck	50	24%	18	32%	33	21%	0.100
Upper extremity	31	15%	10	18%	22	14%	0.503
Lower extremity	61	29%	23	41%	39	25%	**0.023**
Spine	61	29%	21	38%	41	26%	0.114
Abdomen	50	24%	21	39%	28	18%	**0.001**
Patient management							
Intubation	31	15%	25	45%	6	4%	**<0.001**
Blood transfusion	42	20%	30	55%	13	8%	**<0.001**
Length of stay, days	8.04±8.53		15.80±11.49		5.25±4.76		**<0.001**

Significant (*p* < 0.05) differences between ICU and non-ICU cohorts are marked in bold font.

Categorical data are expressed as n (%); continuous variables as mean ± SD.

**Figure 1:**
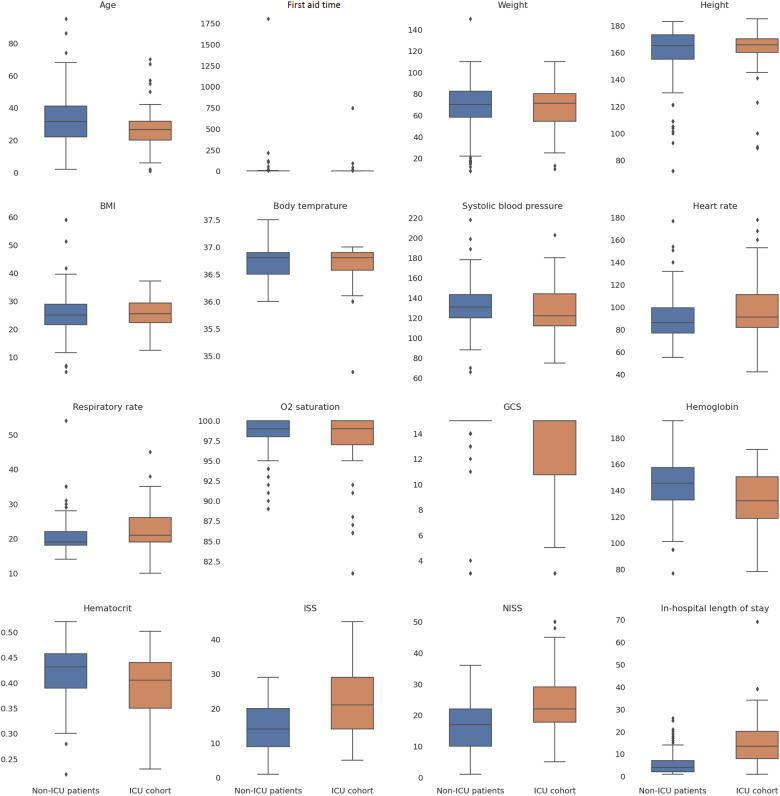
Variations in clinical and laboratory parameters in ICU and non-ICU patients.

According to the results in anatomical scoring systems and GCS, ICU cases were more severe than the others. GCS scores were significantly lower in the ICU group suggesting a more altered consciousness compared to non-ICU patients (12.54±3.79 vs. 14.65±1.67, p<0.05). ISS and NISS indicated a greater severity of injuries in patients under intensive therapy. The ICU cohort had considerably higher AIS scores for the head, face, abdomen, spine, and lower extremity. The traumas were minor in both groups according to the scoring system. Thorax injuries can be categorized as severe (AIS = 3). However, the difference in scores was not significant between ICU and non-ICU patients. Variation of laboratory findings and clinical parameters is illustrated in Figure 1.

A notably greater portion of ICU patients had surgical emphysema compared to the non-ICU cohort (p=0.031). The percentage of lower extremity and abdomen injuries was substantially larger in ICU patients (p=0.023 and p=0.001 respectively). These findings correspond to the inter-group difference in AIS scores for Abdomen and Lower Extremity.

A significantly greater portion of ICU-patients required intubation and blood transfusion compared to non-critical patients. The non-ICU cohort had a nearly three times shorter length of in-hospital stay compared to ICU patients (5.25±4.76 vs. 15.8±11.47, p<0.001).

### 4.2 Prediction of ICU admission

**Figure 2:**
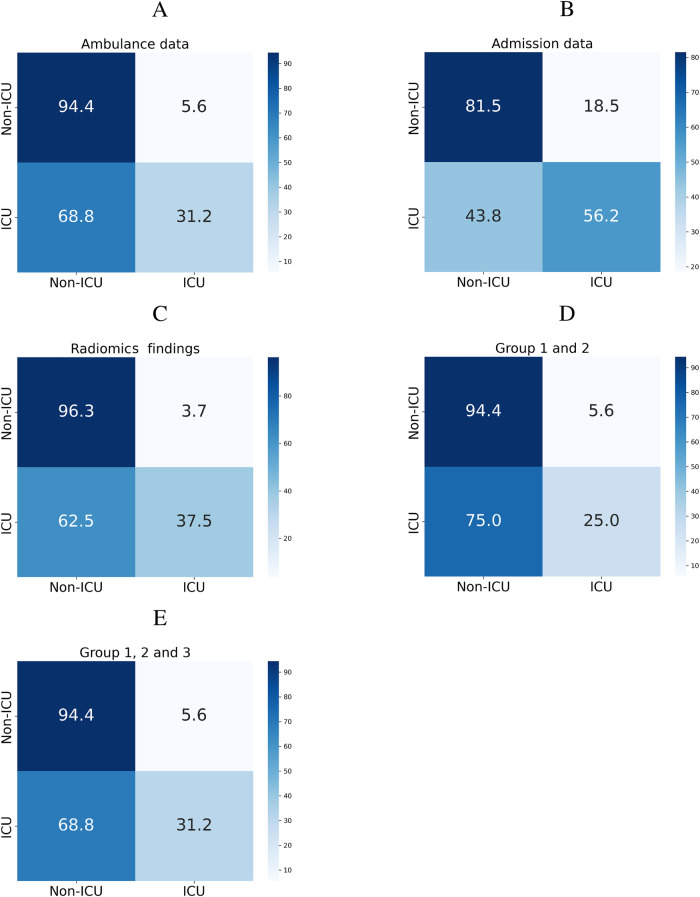
Classification matrices showing performance of models predicting ICU admission from ambulance data (**A**), admission findings (**B**), radiomics (**C**), and their combinations (**D**,**E**).

To predict whether the patient will require intensive care, we trained a machine learning classification model on three groups of findings collected at different time points (see Figure 2). When trained on the ambulance data, the models produced borderline performance: the patients who wouldn’t worsen were identified with 81.5% sensitivity. We retrained the models on a combination of the ambulance data, results in laboratory tests, findings in physical and radiologic examinations. The accuracy improved reaching the sensitivity level of 94.4%. If trained on the radiomics data, the classification algorithms showed the optimal performance with 96.3% sensitivity.

**Figure 3:**
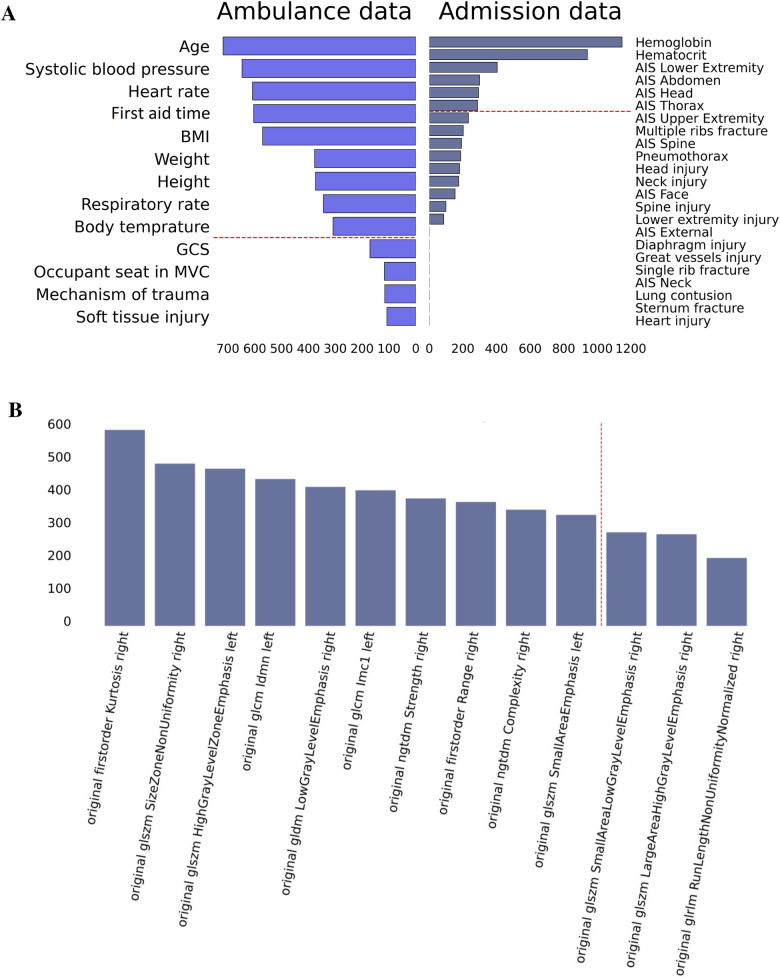
Feature importance in prediction of ICU transfer: data on admission, ambulance data **(A)**, and radiomics findings **(B)**.

The top risk factors were extracted with a feature selection technique (see Figure 3). From the data collected in the ambulance, the best-performing features were age, vital signs, anthropometrics, and first aid time. From the admission data, the top-rank predictors were the laboratory findings, AIS scores for the lower extremity, abdomen, head, and thorax. The original first-order kurtosis had the highest predictive value among the radiomics data. The top-informative radiomics data were derived from the right hemithorax. Presumably, the contribution of the right side estimates to the prognosis is higher because the right lung is larger.

### 4.3 Prognostication of in-hospital length of stay

**Figure 4:**
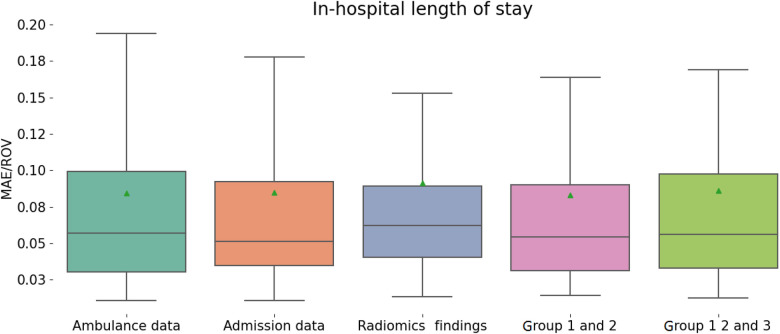
Performance of regression models in predicting in-hospital length of stay from different groups of variables.

Accurate prognosis on the duration of hospitalization can improve patient management by recognizing health issues, optimizing treatment plans, allocating hospital resources and medical staff among patients. It also contributes to tracking the recovery. We constructed regression models that reliably reflect the LOS of the patients (see Figure 4 and Table 4). For this, the following predictors were used: demographics, clinical risk scores, radiomics data, laboratory and radiologic findings as well as their combinations. When trained on different groups of data, the machine-learning regression models showed similar performance with MAE/ROV around 8÷9%.

**Table 4:** Performance of regression models predicting in-hospital length of stay.

Model	Ambulance data		Admission data		Radiomical findings		Group 1 and 2		Group 1, 2 and 3	
	MAE	MAE/ROV	MAE	MAE/ROV	MAE	MAE/ROV	MAE	MAE/ROV	MAE	MAE/ROV
CatBoost Regressor	5.68±5.59	0.08±0.08	5.84±5.85	0.09±0.09	6.23±5.58	0.09±0.08	5.50±5.62	0.08±0.08	5.86±5.71	0.08±0.08
Random Forest	5.56±5.97	0.08±0.09	5.55±6.39	0.08±0.09	6.05±6.38	0.09±0.09	5.32±5.79	0.08±0.08	5.76±6.29	0.08±0.09
LGBM Regressor	5.96±5.62	0.09±0.08	5.89±5.39	0.09±0.08	6.23±5.52	0.09±0.08	5.76±5.19	0.08±0.07	6.04±5.62	0.09±0.08
XGB Regressor	5.78±6.69	0.09±0.09	5.43±6.12	0.08±0.09	6.50±6.82	0.09±0.10	5.59±6.17	0.08±0.09	5.92±6.76	0.09±0.10
Decision Tree Regressor	5.76±6.37	0.09±0.09	6.10±6.69	0.09±0.09	6.10±6.69	0.09±0.10	6.10±6.69	0.09±0.09	5.76±6.37	0.08±0.09
Mean ± SD	5.75±6.05	0.08±0.09	5.77±6.09	0.08±0.09	6.23±6.20	0.09±0.09	5.65±5.89	0.08±0.08	5.86±6.15	0.09±0.09

**Figure 5:**
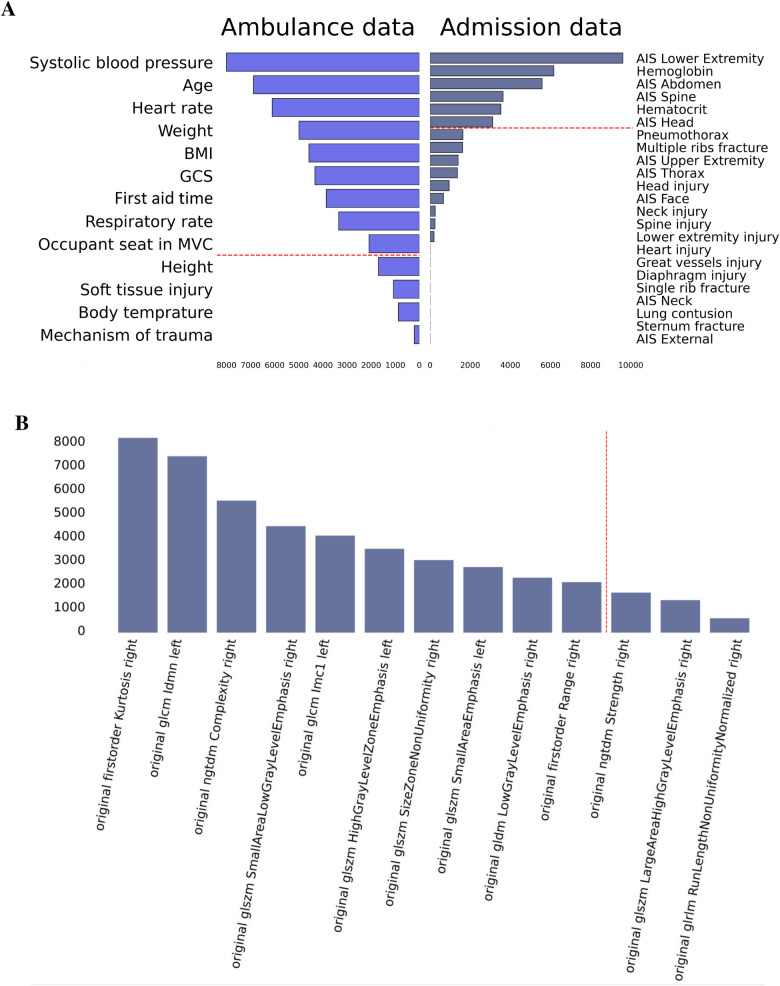
Feature importance in prediction of in-hospital LOS: data on admission, ambulance data **(A)**, and radiomics findings **(B)**.

We used feature engineering to improve model performance, explore how the model works, and increase the clinician’s trust in the model that otherwise looks like a “black box” solution. In our study, the clinical scores describing thoracic trauma were weak predictors of the LOS. Contrarily, the anatomic scores for other body parts and laboratory markers of hemorrhage were strong correlates of the targeted variable. AIS_*lower_extremity*, laboratory findings, AIS_abdomen, AIS_spine, and AIS_head were top informative predictors of the in-hospital stay (see Figure 5). Hence, the higher the number of injured body parts the more severe the case is.

## 5 Discussion

The prevalence of BCT in the studied population was 0.69^0^/_0000_. Information on other populations and countries is scarce. For instance, a study in New Zealand reported a higher incidence of thoracic injury (2.49^0^/_0000_) since it included both blunt and penetrating injuries of the thorax (30). The statistics on penetrating injuries were missing for our population, which did not allow us to perform a comparative analysis.

In our study, traffic accidents accounted for most BCT traumas (69%). We also considered injuries caused by falls from a height of over one meter (18%). Only 26% cases admitted to the hospital were transferred to the ICU. We observed a pronounced between-group difference in AIS scores for head, face, abdomen, spine, and lower extremity damages (p<0.05). The ICU cohort had higher chances of developing surgical emphysema. Patients who required intensive care stayed in the hospital three times longer (15.8±11.47 vs. 5.25±4.76, p<0.001).

Commonly, thoracic injuries are studied in the context of polytraumas (31–33). Risk assessment in severe chest monotrauma is also an issue of ongoing studies. Researchers investigate the effect of severe chest injury on mortality in trauma patients admitted to the ICU (32). The number of articles on BCT is lower than those on traumas in other anatomic regions. Contrarily, we focused on moderate to severe thoracic trauma (AIS_thorax=3.0±0.91) to assess the impact of BCT on patients’ outcomes.

### 5.1 Statistics on ICU patients with BCT

Herein, we describe patient profiles of those requiring intensive care for BCT. Trauma management starts with history taking. In our study, 65.5% of the cases hospitalized in the ICU sustained their injuries in the street. The most common mechanisms of trauma were motor vehicle collisions (MVCs) such as car crashes and pedestrian accidents. Other authors also identified MVCs as the most common cause for BCT (33–35). Falls and criminal activity were other leading reasons for these injuries (36–38).

The assessment of the clinicodemographic profile is the next step in risk stratification. Therefore we included clinicodemographic features in the analysis of the risks of ICU admission. In other studies, researchers focused on trauma complications that developed after admission to the ICU. For example, Lin et al. distinguished complications between patients requiring standard and prolonged ICU stay. The latter was associated with a head injury, multiple rib fractures, chest drain placement, spinal surgery, and extremity surgery (39). Ekpe et al. compared deceased and survived BCT patients and identified the delayed presentation to the emergency department as a determinant of morbidity and mortality (40). As these authors did not consider clinicodemographic parameters, this may reduce the performance of their models.

We also studied laboratory predictors of patients’ outcomes in BCT. In our dataset, ICU patients had a lower concentration of hemoglobin and hematocrit in the blood compared to the non-ICU cohort. However, the average values in each group were within the normal range, since laboratory findings may not reflect the case severity. A study on pneumothorax showed minor differences in the red blood cell parameters between the patients with and without air in the pleural cavity. The parameters also ranged within the reference norm (41). Another study on BCT reported a drop in hemoglobin and hematocrit. Still, the levels did not vary across groups of patients with different chest AIS and ISS (42). As seen from our findings and the findings of our colleagues, the reliability of laboratory predictors in forecasting BCT outcomes is questionable.

Injuries to other parts of the body may aggravate the patient’s status. Hildebrand et al. observed the multi-organ dysfunction syndrome or sepsis in 14% of BCT patients with the AIS_thorax of 3 and above (35). The BCT death rate varies significantly across studies. The proximity of the heart and big vessels to the hemithorax results in their injury, complicates the prognosis of BCT, and leads to conflicting statistics from studies in this category of patients (43). A variation in socio-demographic parameters may also account for discrepant findings reported in the literature (42, 44).

### 5.2 Reliability of machine learning in predicting trauma outcomes: ICU admission and in-hospital LOS

We applied a modern statistical approach to stratify patients into severe and mild-to-moderate cases. Specifically, we trained machine learning (ML) models prognosticating the in-hospital LOS and clinical worsening which would require intensive care. In this technique, we identified top informative predictors from a set of data collected by the ambulance team, hospital staff of the admission yard, and the bioengineers who performed radiomics analysis of CT imaging findings. A strength of our research is the fact that we considered a large amount of data including radiomics (45–48).

Staziaki et al. also programmed ML algorithms to predict ICU admission in patients with torso trauma (45). The authors trained classification models on clinical findings and CT imaging data separately and in combination. The best performance was achieved with the Support Vector Machine classifier trained on a combination of radiologic and clinical findings (AUC 0.87±0.03). Artificial neural networks (ANN) were the system architecture of the models trained on raw CT data (AUC 0.81±0.06). In contrast to this study, we resorted to radiomics and analyzed a big set of numeric data instead of imaging findings. This allowed us to construct reliable classification and regression models reflecting the disease course.

Recent studies focused on the optimal predictors of ICU admission after injuries. For example, research on general trauma showed that a motor component of the GCS is the most critical indicator in predicting the optimal use of ICU services in these patients (46). In this research, models reached reputable performance (ROC 0.883÷0.945, accuracy over 90%), and the ANN models trained on clinicodemographic parameters outperformed logistic regression models. In another study on traumatic thoracic injury, the top risk factors of ICU admission were ISS 16, hemothorax, chest tube placement, head, abdominal, and spinal injury (39). In our study, some of these risks served as reliable predictors of the duration of hospitalization after BCT.

In the current research, the high accuracy of regression models was reached due to the thorough analysis of data collected at each time point: ambulance transportation, hospital admission, and radiologic examination. Still, age-related risks and concomitant trauma are the major factors that determine the duration of in-hospital treatment. Specifically, systolic blood pressure and AIS for lower extremities are top informative predictors according to our data. In another study, systolic blood pressure over 90 was also associated with the ICU stay after severe chest trauma (47). Low arterial pressure indicates a high risk of mortality in severe thorax injuries (36).

Another research reported the parameters linked with the in-hospital LOS over 6 days in patients with any type of thorax trauma. These are female gender, ICU hospitalization, oxygen supplementation or mechanical ventilation at treatment, and ISS scores (48). We did not consider these parameters as predictors since most of them were unknown at hospitalization.

### 5.3 Chest injury in polytrauma

As we focused on the injury to the chest, the studied cases fell under the criteria of mono- rather than polytrauma. Still, the majority of patients had injuries to other body parts: the head, lower extremities, and spine. Concomitant injuries were also reported in other studies on BCT (6, 32, 36, 38). Authors showed substantial concomitant damage to the head and extremities in 30% and 20% of BCT cases, respectively (32, 36). The injury to other body parts can aggravate BCT.

From our data, MVC accounts for the majority of BCT cases. In car accidents, MVC-associated forces directly or indirectly injure the body (49). The chest collides with the steering wheel or airbag, and the head may hit the windshield (36). A car crash test protocol contains airbag deployment, head impact, injury testing, seat belt strength and pull testing. However, crash-testing engineers should pay particular attention to the contact between the chest and the steering wheel, since our statistics show that the highest proportion of BCT patients occupy the driver seat (29%).

Although the issue of the study was chest trauma, we failed to find a significant difference in AIS_thorax between ICU- and non-ICU patients (3.12±0.79 vs. 2.96±0.95; p=0.367). However, the indices for the head and face exhibited a pronounced difference between the two groups of patients: 1.16±1.72 vs. 0.43±0.89, **p=0.009** and 0.45±0.76 vs. 0.19±0.50, **p=0.017**, respectively. A recent study on BCT also reported high mortality due to concomitant head trauma (32, 34). In the context of polytrauma, patients with AIS_thorax of 3 and above may die from respiratory complications (50). For this reason, patients with multiple traumas and high severity of chest injury are candidates for ICU admission (33, 34) and pre-hospital intubation (51).

### 5.4 Importance of radiological findings for optimal patient management

Our findings can be used to optimize the management of patients with a chest blunt injury. The models we built may help physicians to stratify BCT patients by risk of worsening and overcome the limitations of existing tools for risk assessment. High-quality AI models trained on radiomics data demonstrate superior performance.

In BCT, the advanced risk stratification requires a radiological examination of the thorax. Chest computed tomography (CT) may minimize the overall treatment costs in this category of patients. CT can detect patchy or nonsegmental alveolar opacities, mixed lesions, and consolidations (52). It can also sensitively identify parenchymal abnormalities including focal or diffuse homogeneous opacification (53). Lung contusion, pneumothorax, or hemothorax are visible in CT images, therefore spiral chest CT is recommended in all trauma patients with multiple trauma and suspected thoracic injuries (54, 55). However, structural damage can be missed in initial CT scans and revealed in the follow-up examination (56).

The extended examination protocol includes the contrast-enhanced study which helps to rule out complications such as acute thoracic aortic injury (57, 58). In polytrauma patients, post-enhanced imaging is suggested to detect injury of the major mediastinal vessels and the heart (59). In BCT cases, helical chest CT identifies traumatic arterial injuries with 73% sensitivity and 100% specificity (60). However, intravenous contrast may cause iatrogenic complications (61).

## 6 Limitations

• A limitation of the study is that we used 3D Slicer, although it is not an FDA-registered tool for the provision of patient care. Still, this software is widely used for research in basic biomedical and clinically applied settings as it offers several advantages (62). First, it is not linked to any specific hardware. Second, it supports versatile visualizations with advanced functionality (e.g., automatic segmentation). Third, with 3D Slicer, we can perform quantitative research in image analysis (63).
• Another limitation is that ICU admission decisions often depend on numerous factors. For instance, there may be a certain degree of subjectivity regarding ICU admission criteria, which can vary between physicians and institutions. Besides, the standards differ among hospitals and clinical settings: some US hospitals admit patients to ICU 20 times more often than others (64). Factors such as ICU occupancy levels could also influence these decisions (64, 65). Other reasons include the level of ICU physician experience and insufficient data on the patient’s condition (66, 67). The LOS may also be affected by non-clinical factors. For example, governmental subsidy affects hospitalization rates in the UAE: private sector hospitals have an average LOS of 1.48 days compared to 14 days for public hospitals (68). Insurance policy is also associated with in-patient claims and cost of treatment (68).
• A weakness of our study is that the data on BMI in the ambulance record might be unreliable. To illustrate, the weight and height of the patients were derived from patient-reported information. For those who could not provide it, the anthropometric findings were reported approximately.

## 7 Conclusion

• We analyzed a total number of 212 cases treated for BCT in a community-based hospital. The hospital provides care to more than eighty percent of trauma patients who need admission, therefore, the data can represent the entire population of the city. The information from other hospitals is missing, therefore the accurate statistics for the city population remain unknown.
• The prevalence of BCT in the studied population was 0.69^0^/_0000_. Most traumas occurred in traffic accidents (69%). We also considered the injuries caused by falls from a height of over one meter (18%). Only 26% of cases admitted to the hospital were transferred to the ICU. We observed a pronounced between-group difference in AIS scores for the head, face, abdomen, spine, and lower extremity damages (p<0.05). The ICU cohort had higher chances of developing surgical emphysema. Patients who required intensive care stayed in the hospital three times longer (15.8±11.47 vs. 5.25±4.76, p<0.001).
• The study findings can be used to optimize the management of patients with a chest blunt injury as a specific case of monotrauma. The models we built may help physicians to stratify BCT patients by risk of worsening and overcome the limitations of existing tools for risk assessment. High-quality AI models trained on radiomics data demonstrate superior performance.
• To predict whether the patient will require intensive care, we used three groups of findings: ambulance, admission, and radiomics data. When trained on the ambulance data, the models exhibited a borderline performance. The metrics improved after we retrained the models on a combination of ambulance, laboratory, radiologic, and physical examination data (81.5% vs. 94.4% Sn). Radiomics data were the top-accurate predictors (96.3% Sn).
• We constructed regression models that can adequately reflect the in-hospital LOS. When trained on different groups of data, the machine-learning regression models showed similar performance (MAE/ROV around 8%). Anatomic scores for the body parts other than thorax and laboratory markers of hemorrhage had the highest predictive value. Hence, the number of injured body parts correlated with the case severity.

## Data Availability

The raw data supporting the conclusions of this article will be made available by the authors, without undue reservation.
